# Mitochondrial Quality Control in Cardiac-Conditioning Strategies against Ischemia-Reperfusion Injury

**DOI:** 10.3390/life11111123

**Published:** 2021-10-21

**Authors:** Wylly Ramsés García-Niño, Cecilia Zazueta, Mabel Buelna-Chontal, Alejandro Silva-Palacios

**Affiliations:** Department of Cardiovascular Biomedicine, National Institute of Cardiology “Ignacio Chávez”, Mexico City 14080, Mexico; ana.zazueta@cardiologia.org.mx (C.Z.); mabel.buelna@comunidad.unam.mx (M.B.-C.); alejandro.silva@cardiologia.org.mx (A.S.-P.)

**Keywords:** myocardial infarction, ischemic preconditioning, ischemic postconditioning, cardioprotection, mitochondrial biogenesis, mitochondrial dynamics, autophagy, mitophagy, apoptosis, exosomes

## Abstract

Mitochondria are the central target of ischemic preconditioning and postconditioning cardioprotective strategies, which consist of either the application of brief intermittent ischemia/reperfusion (I/R) cycles or the administration of pharmacological agents. Such strategies reduce cardiac I/R injury by activating protective signaling pathways that prevent the exacerbated production of reactive oxygen/nitrogen species, inhibit opening of mitochondrial permeability transition pore and reduce apoptosis, maintaining normal mitochondrial function. Cardioprotection also involves the activation of mitochondrial quality control (MQC) processes, which replace defective mitochondria or eliminate mitochondrial debris, preserving the structure and function of the network of these organelles, and consequently ensuring homeostasis and survival of cardiomyocytes. Such processes include mitochondrial biogenesis, fission, fusion, mitophagy and mitochondrial-controlled cell death. This review updates recent advances in MQC mechanisms that are activated in the protection conferred by different cardiac conditioning interventions. Furthermore, the role of extracellular vesicles in mitochondrial protection and turnover of these organelles will be discussed. It is concluded that modulation of MQC mechanisms and recognition of mitochondrial targets could provide a potential and selective therapeutic approach for I/R-induced mitochondrial dysfunction.

## 1. Introduction

Myocardial ischemia/reperfusion (I/R) injury is a clinical condition associated with myocardial infarction that can cause ventricular arrhythmias, contractile dysfunction and mortality [1]. Blockage of blood flow to the myocardium (ischemia) deprives cardiomyocytes from oxygen and nutrient supplies necessary to oxidize energetic substrates, inhibiting ATP synthesis by mitochondria and promoting cell death [2]. Paradoxically, crucial restoration of blood flow (reperfusion) by primary percutaneous coronary intervention or with thrombolytic drugs aggravates tissue damage due to many pathophysiological mechanisms [3], especially as a consequence of the exacerbated generation of reactive oxygen and nitrogen species (ROS/RNS) that promote a highly oxidative cellular environment, affecting redox-sensitive cellular components, inducing further cardiomyocyte death and affecting other types of cells such as fibroblasts, endothelial cells or smooth muscle cells [4].

Mitochondria represent approximately one-third of cardiac mass and are responsible for cellular energy production via oxidative phosphorylation (OXPHOS) [5]. Additionally, these organelles exhibit great metabolic flexibility, regulating redox signaling, cytosolic Ca^2+^ buffering capacity, immune responses, and programmed cell death, whose dynamic regulation is essential under physiological conditions and is frequently altered in disease [6]. Mitochondria are very sensitive to low oxygen levels during ischemia, as well as ROS generated during reperfusion, which causes loss of mitochondrial function and makes cardiomyocytes susceptible to the deleterious effects of I/R injury [7].

**Figure 1:**
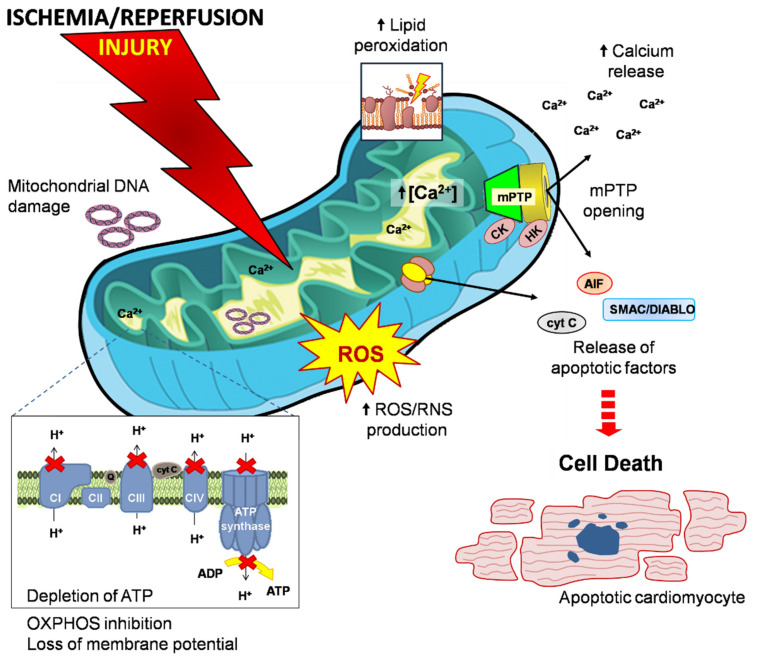
Ischemia/reperfusion-induced mitochondrial dysfunction is characterized by increased generation of reactive oxygen and nitrogen species (ROS/RNS), oxidative stress, impaired oxidative phosphorylation (OXPHOS), depolarization of the transmembrane potential, depletion of adenosine triphosphate (ATP) reserves, mitochondrial Ca^2+^ overload, activation of the mitochondrial permeability transition pore (mPTP) and the release of apoptotic factors. AIF, apoptosis-inducing factor; CI-CIV, complexes I-IV; CK, creatine kinase; cyt C, cytochrome C; DIABLO, direct inhibitors of apoptosis (IAP)-binding protein with low pI; HK, Hexokinase; SMAC, second mitochondria-derived activator of caspase.

Therefore, mitochondrial damage and subsequent dysfunction have been characterized as the hallmark of I/R injury and precursors of cell death caused by the irreversible opening of a nonspecific pore in the inner mitochondrial membrane (IMM), known as the mitochondrial permeability transition pore (mPTP) (Figure 1) [8]. Opening of mPTP leads to the loss of mitochondrial membrane potential (ΔΨm), disruption of OXPHOS and the consequent depletion of adenosine triphosphate (ATP), as well as osmotic shock to the mitochondria, rupture of the outer mitochondrial membrane (OMM) and cardiomyocyte death [9].

Given the relevance of mitochondria against I/R damage and the central role they play in cell homeostasis, one of the main objectives of cardioprotective strategies should be to limit or nullify I/R-induced mitochondrial alterations through the activation of quality control mechanisms to obtain a greater survival of cardiomyocytes, avoid the progressive decline of surviving myocytes and improve the functionality of the ischemic tissue. Thus, this review describes the latest findings regarding the mitochondrial quality control (MQC) mechanisms that are activated by cardiac conditioning strategies: ischemic preconditioning (IPC) and ischemic postconditioning (iPostC), which activate endogenous mechanisms that make the myocardium more tolerant against reperfusion injury [10]. Additionally, extracellular vesicles (EVs) have been shown to contribute to the protection exerted by these cardioprotective maneuvers [11]. For that reason, we will discuss the relevance of EVs and their emergence as critical actors for mitochondrial protection and/or mitochondrial renewal in response to IPC and iPostC.

## 2. Protecting the Mitochondria: The Key Target in Myocardial Ischemic Conditioning

IPC and iPostC are two cardioprotective strategies that successfully reduce lethal I/R injury [12,13]. IPC involves the mechanical application of brief episodes of ischemia and reperfusion before sustained ischemia, whereas in iPostC these episodes are applied after sustained ischemia and at the beginning of reperfusion [14]. Additionally, interventions that mimic myocardial ischemic conditioning have been developed, including the use of pharmacological agents (pharmacological conditioning) [15], mechanical and intermittent interruptions in blood flow of a distant organ or tissue (remote ischemic conditioning, RIC) [16], the periodic application of hypoxic gas mixtures or intermittent high altitude hypoxia (hypoxic conditioning, HypC) [17,18], among others [19,20].

Several studies in animal models have demonstrated that IPC and iPostC limit cardiac damage, reduce infarct size and promote recovery of cardiac mechanical function by modulating pH and decreasing the amount of harmful ROS [21,22]. Both strategies activate protective signal transduction pathways that converge in mitochondria [13,23], inhibiting mPTP opening and promoting cardiomyocyte survival [24].

IPC elicits a bi-phasic pattern of cardioprotection. The first phase begins immediately after the IPC and is extended for 1–2 h (early IPC), while the second phase appears 12–24 h later and lasts for 48–72 h (delayed or late IPC) [21]. In particular, IPC protects the myocardium by inhibiting mPTP opening and activating mitochondrial ATP-sensitive K^+^ (mitoK_ATP_) channels [25,26]. Moreover, IPC preserves mitochondrial respiratory function and promotes mitochondrial hyperpolarization (increased ΔΨm), which augments ATP generation and respiratory capacities, improving energy production and counteracting the loss of ATP induced by anoxia-reoxygenation [27]. Additionally, IPC prevents cytochrome C release and apoptosis [28], whereas it preserves mitochondrial ultrastructure and reduces mitochondrial protein oxidation in ex vivo and in vivo models [29]. In vitro studies have contrasted and provided complementary information to that obtained in animal models. For example, pharmacologic preconditioning in cardiomyocytes preserves cell viability, maintains ΔΨm, ATP synthesis and antioxidant response after hypoxia/reoxygenation (H/R); conversely, ROS production, Ca^2+^ overload, cytochrome C release and apoptosis are inhibited [30,31,32].

On the other hand, iPostC promotes acidosis and gradual oxygenation, which are necessary to generate ROS signaling and interfere with mPTP formation and opening [22,33]. Notably, the opening of mitochondrial Ca^2+^-activated K^+^ (mitoK_Ca_) and mitoK_ATP_ channels was also related to the protective effects of iPostC in rabbit and rat hearts [34,35]. iPostC preserves mitochondrial ultrastructure and cardioprotection has also been associated with decreased mitochondrial oxidative stress and increased buffering capacity to overcome Ca^2+^ overload and cytochrome C release in rat hearts subjected to I/R [24,36]. Sevoflurane postconditioning (SPostC), a commonly used anesthetic, reproduces all of the above effects in both ex vivo and in vivo models [12,37,38,39,40,41], and also maintains mitochondrial morphology and preserves mitochondrial dynamics [42].

Currently, numerous studies are being conducted to understand how IPC and iPostC promote mitochondrial turnover, which ensures cardiomyocyte homeostasis and survival [43,44], as will be reviewed below.

## 3. Mitochondrial Quality Control in Cardioprotection

**Figure 2:**
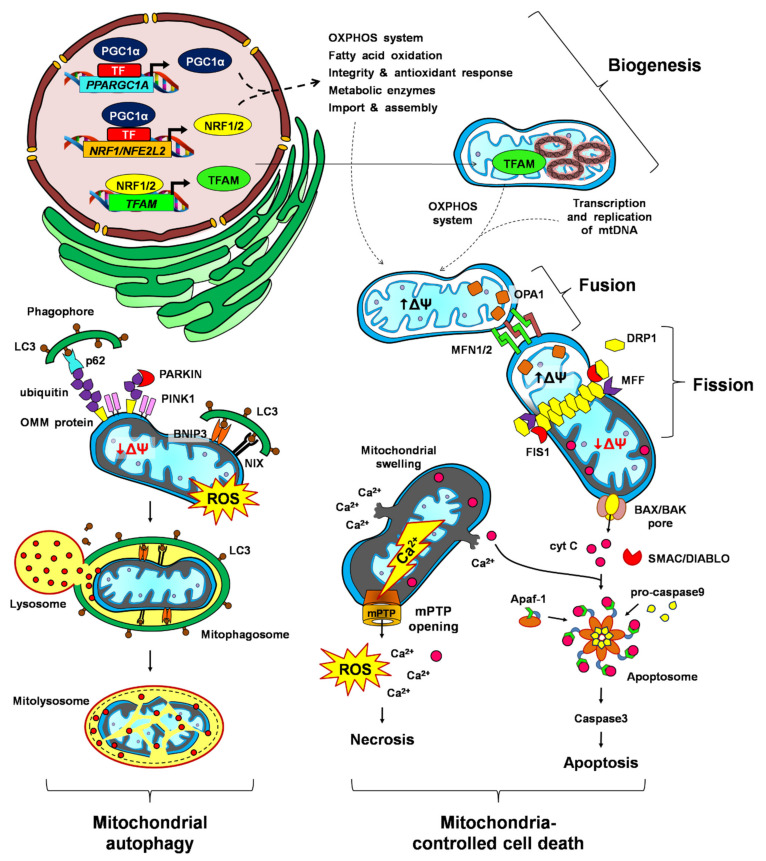
Mitochondrial quality control (MQC) maintains a highly efficient population of mitochondria and preserves cellular homeostasis through complex molecular machineries and regulatory proteins. New mitochondria are created through the coordinated expression of genes on both nuclear and mitochondrial genome to replace dysfunctional mitochondria. Mitochondrial fusion occurs when the outer and inner mitochondrial membranes of two adjacent mitochondria fuse and share content, improving mitochondrial function and facilitating communication, while mitochondrial fission splits a mitochondrion into two daughter mitochondria, leading to cell removal of damaged mitochondria. Mitophagy selectively removes depolarized mitochondria for lysosomal degradation. Finally, mitochondria-dependent cell death involves interconnected processes that may occur simultaneously to lead cell death, including necrosis, apoptosis, pyroptosis and necroptosis. A detailed description of the pathways can be found throughout text. ΔΨm, mitochondrial membrane potential; Apaf-1, apoptosis protease-activating factor-1; ATP, adenosine triphosphate; BNIP3, BCL-2/adenovirus E1B 19 kDa protein-interacting protein 3; cyt C, cytochrome C; DIABLO, direct inhibitors of apoptosis (IAP)-binding protein with low pI; DRP1, dynamin-related protein 1; FIS1, mitochondrial fission 1; LC3, microtubule-associated proteins 1A/1B light chain 3B; MFF, mitochondrial fission factor; MFN, mitofusin; mPTP, mitochondrial permeability transition pore; mtDNA, mitochondrial DNA; NIX/BNIP3L, BNIP3-like; NRF, nuclear respiratory factor; OMM, outer mitochondrial membrane; OPA1, optic atrophy protein; OXPHOS, oxidative phosphorylation; p62, sequestosome 1; PGC-1α, proliferator-activated receptor gamma coactivator-1alpha; PTEN-induced kinase 1 (PINK1), PTEN-induced kinase 1; ROS, reactive oxygen species; SMAC, second mitochondria-derived activator of caspase; TF, transcription factor; TFAM, mitochondrial transcription factor A.

Cells have developed protective quality control systems in response to different stresses in order to maintain function [45], including self-regulating processes that detect damaged or abnormal organelles/macromolecules and the activation of repair or degradation mechanisms [46]. The number, ultrastructure and location of mitochondria are critical to satisfy the energetic and metabolic needs of the cell [47]. Therefore, these organelles are subject to finely regulated MQC mechanisms, which respond to stress stimuli and activate a network of pathways that act dynamically to maintain a highly efficient population of mitochondria [5]. MQC mechanisms include (i) mitochondrial biogenesis, which involves the expansion of mitochondrial numbers; (ii) mitochondrial dynamics (fusion and fission), which allows the redistribution and exchange of mitochondrial components among the mitochondrial population; (iii) mitochondrial autophagy (mitophagy), related to the selective degradation of mitochondria; and (iv) mitochondria-controlled cell death (Figure 2) [48,49].

The imbalance between the processes that govern mitochondrial turnover results in the accumulation of dysfunctional mitochondria, an aberrant cellular response, and leads to pathologies, such as acute myocardial infarction and heart failure [50,51]. We refer the reader to some of the more recent reviews that have focused in depth on the relevance of MQC in cardiac health and disease [5,52,53,54].

## 4. Mitochondrial Biogenesis during Conditioning: It Is Not Too Late to Renew

### 4.1. Mitochondrial Biogenesis

Mitochondrial biogenesis is a highly regulated process by which new mitochondria are produced from existing ones, leading to an increase in mitochondrial mass and maintenance of population turnover and cellular energy [55]. This process requires the coordinated expression of genes on both the nuclear and mitochondrial genomes, along with the de novo synthesis of mitochondrial DNA (mtDNA) and lipid components of the membrane [56].

A murine cardiac proteomic study revealed that mitochondria contain about 4906 proteins located in different mitochondrial compartments [57]. Most of these proteins are encoded by the nuclear genome and are imported into the mitochondria through multi-subunit protein complexes, named the translocase of the outer membrane (TOM), and the inner-membrane translocases (TIM), e.g., TIM23 and TIM22 complexes [58]. Only 13 subunits of respiratory complexes I, III, IV, and V, along with two ribosomal and 22 transfer RNAs, are encoded by the circular and double-stranded mtDNA [59].

The nuclear-encoded peroxisome proliferator-activated receptor gamma (PPARγ) coactivator-1alpha (PGC-1α) is the master regulator of mitochondrial biogenesis [60]. PGC-1α is activated for its translocation to the nucleus via phosphorylation by AMP-activated protein kinase (AMPK) or deacetylation mediated by NAD^+^-dependent protein deacetylase sirtuin 1 (SIRT1) [61]. In the nucleus, PGC-1α binds and activates different transcription factors, including the nuclear respiratory factors 1 and 2 (NRF1 and NRF2) [62], the estrogen-related receptors (ERRs) [63], the forkhead box class-O1 (FOXO1) [64], and PPARγ [65]. In turn, these factors stimulate the transcription of mitochondrial enzymes involved in OXPHOS, fatty acid β-oxidation, mitochondrial import and export systems, antioxidant defense and mtDNA synthesis. Some of them are assembled into larger complexes with proteins encoded by mtDNA within the newly synthesized inner and outer mitochondrial membranes [66]. Furthermore, NRF1 and NRF2 activate mitochondrial transcription factor A (TFAM), which is a mtDNA-binding protein essential for genome maintenance and responsible of mtDNA transcription, replication, repair and packaging [67].

### 4.2. Regulation of Mitochondrial Biogenesis by Cardiac Conditioning

Impaired mitochondrial biogenesis has been observed in myocardial infarction [68], cardiac hypertrophy [69], dilated cardiomyopathy [70] and heart failure [71]. In hearts subjected to I/R, the mtDNA copy number is drastically reduced, together with diminished p-AMPK, PGC-1α, peroxisome proliferator-activated receptor alpha (PPARα) and TFAM protein levels [72,73], as well as cytochrome C oxidase subunit 4 (COX4) and TOM70 mRNA levels [74]. Additionally, H/R inhibits the AMPK pathway and downregulates PGC-1α, NRF1, TFAM, TOM20, TIM23, nuclear factor erythroid 2-related factor 2 (Nrf2) and sirtuin 3 (SIRT3) levels, in association with altered mitochondrial biogenesis, mitochondrial dysfunction, augmented oxidative stress, and apoptosis in cardiomyocytes [75,76]. Similarly, mitochondrial biogenesis is disrupted in cardiac microvascular endothelial cells after simulated I/R injury [77].

McLeod et al. (2004) reported the first evidence that delayed IPC modulates mitochondrial biogenesis after determining the upregulation of NRF1 and PGC-1α in parallel with the activation of nuclear genes that encode mitochondrial proteins, i.e., succinate dehydrogenase, cytochrome C oxidase, cytochrome C and adenine nucleotide translocase-1 (ANT1). This effect was accompanied by preserved activities of the electron-transfer chain and OXPHOS [27]. A newly described component of the IPC pathway that promotes mitochondrial biogenesis is the stress-response protein GJA1-20k, which was increased in both total heart lysates and in mitochondria from ex vivo mouse hearts subjected to this conditioning maneuver [78]. GJA1-20k is an alternatively translated isoform of Connexin 43 (Cx43) that acts as a Cx43 trafficking chaperone, promotes microtubule-dependent mitochondrial transport and regulates gap junction formation and mitochondrial function [79,80]. Administration of exogenous GJA1-20k via an AAV9 gene delivery system to mouse hearts also activates transcriptional pathways that promote mitochondrial biogenesis, increasing mtDNA copy number and enhancing the expression of PGC-1α and cytochrome C oxidase subunit 2 (COX2) [78]. The GJA1-20k study is gaining relevance as a cardioprotective target due to its potential to maintain the integrity of the mitochondrial network during cellular stress.

Surprisingly, the effect of iPostC on the regulation of mitochondrial biogenesis is a field that has received little attention and needs to be explored. However, some evidence suggests a positive regulation of iPostC on mitochondrial biogenesis. For example, hypoxic postconditioning (Hyp-PostC) improves post-ischemic cardiac function, restores ATP content, and increases PGC-1α mRNA levels in isolated perfused rat hearts [81]. Another novel cardioprotective strategy in rats called therapeutic hypercapnia, which consists of the inhalation of 20% CO_2_ after ischemia, has been used in the rat model of coronary artery ligation. It mimics iPostC acidosis, ameliorating mitochondrial morphological damage and dysfunction, along with PGC-1α and TFAM mitochondrial biogenesis pathway upregulation [73].

Pharmacological conditioning has provided more information on the activation of biogenesis pathways. In this regard, resveratrol preconditioning, a potent polyphenolic antioxidant that protects the heart against I/R injury [82], regulates mitochondrial biogenesis, increasing PGC-1α activation and regulating upstream signaling pathways such as SIRT1, nitric oxide (NO), AMPK or p38 mitogen-activated protein kinase (MAPK), among others [83]. In addition, resveratrol promotes the expression of other genes involved in mitochondrial biogenesis, such as NRF1, TFAM and COX4 [84,85]. Melatonin, another molecule with antioxidant properties, promotes mitochondrial biogenesis by activating the AMPK pathway and inducing PGC-1α, TFAM, TOM20, TIM23, Nrf2 and SIRT3 upregulation in cardiomyocytes subjected to H/R. Such activation was related to preserved mitochondrial function and apoptosis inhibition. Conversely, cardiomyocytes transfected with PGC-1α siRNA lose the protection induced by melatonin [75]. Thus, it is probable that antioxidants capable of modulating PGC-1α during cardiac conditioning may have important implications in the regulation of mitochondrial biogenesis.

On the other hand, preconditioning and postconditioning with the histone deacetylase (HDAC) inhibitor suberoylanilide hydroxamic acid (SAHA, Vorinostat) protects mouse hearts and cardiomyocytes against I/R injury. SAHA stimulates mitochondrial biogenesis and promotes the elimination of damaged mitochondria, as well as the reduction in ROS production and mtDNA damage. This compound increased mtDNA levels and mitochondrial mass by inducing the expression of PGC-1α, an effect that was completely abolished by knocking down PGC-1α [86]. Similarly, homoisoflavanone sappanone A applied after ischemia protects rat hearts in an ex vivo reperfusion model and increases mtDNA copy number and PGC-1α expression [43]. Additionally, SPostC upregulates the expression levels of PGC-1α and NRF1 in a model of LAD occlusion, as a compensatory response to I/R-induced mitochondrial dysfunction [12]. Importantly, the cardioprotective neurotransmitter and pharmacological agent acetylcholine protects H9c2 cells against H/R injury when cells are treated with this agent at the beginning of reperfusion. Acetylcholine postconditioning prevents mitochondrial morphologic abnormalities and improves mitochondrial density, mass and function. Moreover, acetylcholine increases mtDNA copy number, activates the AMPK pathway and stimulates upregulation of genes involved in mitochondrial biogenesis, such as PGC-1α, NRF and TFAM. Conversely, knockdown of PGC-1α or AMPK blocks the effects of acetylcholine [87]. The pharmacological use of these agents to prevent myocardial infarction by maintaining the mitochondrial population is a goal for future translational research.

## 5. Fusion and Fission: Two Dynamic Processes in Constant Equilibrium

### 5.1. Mitochondrial Dynamics

Mitochondria undergo coordinated cycles of fusion and fission to control their number, distribution and morphology [88]. These processes are important for mitochondrial inheritance, cell cycle regulation, maintenance of mitochondrial functions, transmission of energy status and are also involved in the control of cell quality [89]. Both processes are governed by complex molecular machinery and are finely tuned by regulatory proteins or posttranslational modifications [88].

#### 5.1.1. Mitochondrial Fusion

Mitochondrial fusion is regulated by three GTPases of the dynamin-related family: mitofusins 1 and 2 (MFN1 and MFN2), located in the OMM, catalyze the hydrolysis of guanosine triphosphate (GTP) to guanosine diphosphate (GDP), and are necessary for the fusion of the outer membrane with adjacent mitochondria, while the optic atrophy protein (OPA1), located in the IMM, mediates the fusion of the inner membrane [90]. MFN1 controls mitochondrial tethering more efficiently than MFN2; however, MFN2 is not only related to mitochondrion–mitochondrion interactions, but also the juxtaposition of mitochondria with the endoplasmic reticulum (ER) and other organelles [91]. Membrane tethering is mediated by the formation of homodimers or heterodimers between two mitofusins in adjacent mitochondria [92]. This association triggers GTP hydrolysis-induced conformational changes, which destabilize the lipid bilayer, facilitating mitochondrial docking, lipid mixing and OMM fusion [93]. On the other hand, OPA1 undergoes constitutive proteolytic processes in which the balanced ratio between the two resulting isoforms is required to maintain normal mitochondrial morphology. Both the long N-terminal transmembrane anchored isoform (L-OPA1) and the short form (S-OPA1) lacking the transmembrane anchors interact with cardiolipin. Using an in vitro membrane approach, it was demonstrated that L-OPA1 binds directly to cardiolipin and GTP hydrolysis drives membrane fusion, while S-OPA1 forms a bridge between the larger isoform and cardiolipin [94]. Under stress conditions, mitochondrial depolarization induces total conversion to S-OPA1, inhibiting fusion and causing mitochondrial fragmentation [95]. Thus, mitochondrial fusion generates an elongated phenotype that helps to maintain mitochondrial respiration and facilitates communication and exchange of contents between mitochondrial compartments, which can buffer transient defects in mitochondrial function [96].

#### 5.1.2. Mitochondrial Fission

This is a process coordinated by the GTPase dynamin-related protein 1 (DRP1), which is recruited from the cytosol to mitochondrial fission sites on the OMM surface where it binds to its receptors, i.e., mitochondrial fission factor (MFF), mitochondrial fission 1 (FIS1), and mitochondrial dynamics protein MID49 and MID51 [97]. Recently, Kalia et al. (2018) showed that GTP binding to DRP1/MID49/51 induces a conformational rearrangement that exposes a network of receptor binding sites, initiating the polymerization of cofilaments that surround the low-curvature mitochondrial tubules. After that, GTP hydrolysis induces the dissociation of MID49/51 receptors, shortening of the filaments and curling of DRP1 oligomers into constricted and closed rings, which initiate the constriction of the inner and outer membranes until mitochondrial division is complete [98]. In addition, DRP1-induced constriction is regulated by the actin-nucleating proteins inverted formin 2 (INF2) and formin-binding protein spire 1C (SPIRE1C), which mediates actin polymerization and subsequent myosin recruitment to ER–mitochondria contact sites [99]. Finally, dynamin 2 (DNM2), a large GTPase, is recruited into the neck of DRP1-mediated mitochondrial constriction where it assembles and terminates membrane scission [88]. Additionally, Ca^2+^ influx initiates and potentiates IMM constriction by activating the mitochondrial big-conductance Ca^2+^-dependent K^+^ channel (mitoBK_Ca_) that mediates mitochondrial bulking and depolarization. Synergistically, the cleavage of L-OPA1 to S-OPA1 and its accumulation in the intermembrane space is required for the regulation of OMM-IMM tethering. These findings suggest that constriction of the internal mitochondrial compartment contributes to efficient DRP1-mediated mitochondrial division [100]. In this way, mitochondrial fission creates disconnected mitochondria that are required for cell division and/or removal of damaged mitochondria through mitophagy during cellular stress conditions [52].

### 5.2. Regulation of Mitochondrial Dynamics by Cardiac Conditioning

Increasing evidence suggests that alterations in mitochondrial dynamics lead to cardiac pathologies such as I/R injury [101], hypertrophy [102], heart failure [103], diabetic cardiomyopathy [104] and cardiorenal syndrome [105]. Fusion and fission activate morphological adaptive responses to stress, but mitochondria particularly undergo extensive fission and reduced fusion during acute I/R injury, generating fragmented dysfunctional mitochondria [106]. Mitochondrial fragmentation cleaves the elongated network of mitochondria into small spheres or short rods, promoting mitochondrial swelling and depolarization, loss of ATP synthesis, respiratory defects, increased ROS production and apoptosis, which contributes to cell death in other cell types and leads to tissue damage [101]. In contrast, blocking the fission machinery protects cardiomyocytes from I/R injury by preventing mitochondrial fragmentation, inducing mitochondrial fusion and autophagy, improving mitochondrial function and decreasing susceptibility to mPTP opening and cell death in different I/R models [107,108,109]. In the heart, simultaneous conditional knockout of both mitofusins results in impaired respiration and abnormal mitochondrial morphology, along with loss of contractile function [110]. Conversely, overexpression of mitochondrial fusion proteins MFN1 or MFN2 augments mitochondrial fusion and protects HL-1 cells against simulated I/R injury [107], whereas hearts deficient in both MFN1 and MFN2 are also protected against acute myocardial infarction due to impaired mitochondria/sarcoplasmic reticulum tethering [111]. In addition, DRP1 overexpression causes mitochondrial fragmentations without cardiac pathology, and very interestingly, MFN1/MFN2/DRP1 triple-knockout mice survive longer and manifest a unique pathologic form of cardiac hypertrophy [112]. In turn, mild overexpression of OPA1 protects against cardiac and cerebral ischemia [113], while the metalloendopeptidase OMA1 (which is activated in response to stress and mediates OPA1 proteolytic processing) plays a central role in mediating ischemia-induced heart failure and cardiac hypertrophy [114]. Therefore, preserving mitochondrial fusion will be essential in cardioprotection strategies to counteract the imbalance in mitochondrial dynamics induced by I/R.

In addition, some posttranslational modifications of fusion proteins and their effects on mitochondrial activity have been reported. For example, PINK1-dependent phosphorylation of MFN2 induces translocation of Parkin to OMM upon depolarization, promoting Parkin-mediated ubiquitination of MFN2 in adult cardiomyocytes [115]. Interestingly, this modification reduces the number of mitochondria and increases the distance between ER and mitochondria [116]. Furthermore, in SIRT3 knockout mice, OPA1 hyperacetylation and mitochondrial fragmentation occur, while its overexpression maintains mitochondrial network and morphology, protecting from doxorubicin-mediated cell death [117], while DRP1 activity increases after its phosphorylation at Ser^616^ and is reduced by phosphorylation at Ser^637^ [109]. In this sense, it has been described that Ca^2+^ accumulation during myocardial I/R activates calcineurin, which in turn dephosphorylates DRP1 at Ser^637^, preventing mitochondrial translocation of DRP1 and initiating fission [118].

In general, the study of proteins related to mitochondrial dynamics is still scarce in cardiac conditioning strategies; however, we present the most relevant ones in the field. For example, IPC has been documented to prevent dissociation of the mitochondria-hexokinase 2 (HK2) complex that confers cardioprotection [119]. Recently, Pereira et al. (2020) reported that the release of mitochondria-bound HK2 modulates the binding of DRP1 and mitochondrial-associated DNM2 protein in mitochondria isolated from animals subjected to IPC [120]. In rats subjected to remote ischemic preconditioning (RIPC), preserved mitochondrial morphology was observed due to increased OPA1 [121]. Furthermore, Le Page et al. (2016) reported that the deficiency of this protein in mice is associated with increased sensitivity to I/R and an imbalance in dynamic mitochondrial Ca^2+^ uptake [122]. Cellier et al. (2016) reported that animals subjected to limb ischemia interspersed by hind limb RIPC show smaller infarct size and less accumulation of DRP1 in the mitochondrial fraction compared to I/R animals, emphasizing that RIPC inhibits mitochondrial fragmentation [121].

On the other hand, SPostC increased the expression of MFN2 and OPA1 in association with the inhibition of mPTP and the maintenance of ΔΨm [42]. In addition, SPostC inhibits DRP1 translocation to mitochondria and attenuates cardiac I/R through induction of the provirus integration site for Moloney murine leukemia virus 1 (PIM-1) kinase that activates DRP1, thus preventing excessive mitochondrial fission. Likewise, cardiomyocytes undergoing the same treatment scheme showed enhanced mitochondrial interconnectivity and elongation in the face of H/R [123]. Interestingly, when the cell is stimulated with high glucose, Mdivi-1 (DRP1 inhibitor) reverses the negative effects of high glucose through increased fusion [124]. Furthermore, pharmacological preconditioning with nitrite in H9c2 cells attenuates cell death after hypoxia due to the inhibition of mitochondrial DRP1 by its phosphorylation through protein kinase A (PKA) [125].

In rats with myocardial I/R injury, preconditioning or postconditioning with the acetylcholinesterase (AChE) inhibitor donepezil reduced infarct size and cardiac arrhythmia [126]. Donepezil administrated before, during or after ischemia attenuated cardiac mitochondrial dysfunction and mitochondrial dynamic imbalance by repressing mitochondrial fission protein DRP1 and enhancing the expression of mitochondrial fusion proteins OPA1 and MFN2 [126]. Moreover, preconditioning with penehyclidine hydrochloride (PHC) protects against acute I/R damage in association with decreased DRP1 and increased MFN1 and MFN2 expression. However, in the long term, changes in these proteins did not correlate with the preservation of cardiac function [44]. On the other hand, treatment with Mdivi-1 before myocardial ischemia results in the lengthening of interfibrillary mitochondria and a reduction in the size of the infarct, which confirms that by preventing mitochondrial fission, the induction of mitochondrial fusion is favored, benefiting the survival of cardiomyocytes [107,127]. On the other hand, the administration of hydralazine, an agent used in the treatment of hypertension and chronic heart failure, not only protects the heart from acute I/R through its antioxidants and anti-apoptotic effects, but if administered at the time of reperfusion, it decreases the size of the infarct by inhibiting mitochondrial fission [128].

Additionally, pharmacological treatment of rat hearts with the mitochondrial fusion promoter M1 prior to and during myocardial ischemia, as well as at the onset of reperfusion, results in cardioprotection, reestablishing mitochondrial dynamics balance and enhancing mitochondrial function [101]. On the other hand, melatonin preconditioning and postconditioning inhibits mitochondrial fission by blocking DRP1 activation and its translocation from the cytosol to the mitochondria in Langendorff perfused rat hearts [129]. In addition, melatonin pretreatment and postconditioning inhibit mitochondrial fragmentation and reestablish the balance of mitochondrial fission/fusion by downregulating DRP1, MFF and FIS1, while MFN1, MFN2 are upregulated in a sirtuin 3 (SIRT3)-dependent manner, or mediated by OPA1 expression in H9c2 cells exposed to anoxia and reoxygenation [130,131]. Finally, postconditioning with sappanone A balances mitochondrial dynamics and enhances mitophagy in an AMPK-dependent manner [43].

Overall, and despite incredible advances in understanding mitochondrial dynamics, we found limited animal studies linking I/R to the regulation of these important processes.

## 6. The Double-Edged Sword of Cardiac Conditioning: “Self-Eating”

### 6.1. Autophagy

Autophagy is a catabolic process mediated by the lysosome-dependent cellular degradation system, which serves to remove non-essential components, excessive or damaged organelles, protein aggregates and other unwanted cytoplasmic elements, to recycle metabolic substrates and maintain cellular homeostasis [132]. The autophagic process begins with the formation of a crescent-shaped double membrane called the phagophore, which requires the recruitment of many autophagy-specific proteins to ER [133]. First, AMPK phosphorylates Unc-51-like autophagy activating kinase 1 (ULK1), while the serine/threonine-protein kinase mammalian target of rapamycin (mTOR) inhibits ULK1 activation [134]. Next, the ULK1 complex produces the nucleation of the phagophore by phosphorylating and activating the class III phosphatidylinositol 3-kinase (PI3KC3)/VPS34 lipid kinase complex, which generates domains enriched in phosphatidylinositol 3-phosphate (PI3P), termed omegasome [135]. These omegasomes function as membrane platforms where the autophagic machinery is recruited for the manufacture of autophagosomes [136]. The autophagic machinery encoded by autophagy-related genes (ATG) orchestrates the different steps of autophagy, leading to phagophore nucleation followed by its expansion [137]. Then, the pool of PI3P engages WD-repeat domain phosphoinositide-interacting proteins (WIPI), which recovers ATG9-positive vesicles of membrane origin (plasma membrane, mitochondria, recycling endosomes, or Golgi complex), and promotes its fusion with the phagophore to initiate lipid transfer for lipidation of ATG8/LC3 (hereafter LC3) [138]. On the other hand, ATG4B protease cleaves the small ubiquitin-like protein LC3 to LC3-I, which is translocated to phagophores through the ubiquitin-activating-like enzymes E1 (ATG7), E2 (ATG3) and E3, where it is conjugated to phosphatidylethanolamine to form LC3-II, which is commonly used as a marker for autophagy [139]. LC3 facilitates engulfment of cargo in autophagosomes by directly binding to LIR-containing autophagy receptors, such as p62, also called sequestosome 1 (SQSTM1) [140]. Moreover, the ATG5/ATG12-ATG16L complex and lipidated LC3 facilitate the elongation of the phagophore membrane, which engulfs the adaptor-mediated ubiquitinated substrates and eventually closes in on itself and fuses to form the double-membrane structure called autophagosome [141]. Finally, the autophagosome fuses with lysosomes to form autolysosomes, whose internal acidic environment and hydrolytic content promote the disruption of the inner membrane and the digestion of its contents for reuse by the cell [142].

### 6.2. Regulation of Autophagy by Cardiac Conditioning

Autophagy may be triggered by energy depletion, osmotic and oxidative stresses, starvation, hibernation or ischemia [127,143]. In myocardial ischemia, autophagy is activated through the AMPK pathway to protect the heart from I/R injury and post-ischemia cardiac remodeling [144]. However, excessive ROS production during reperfusion impairs autophagy by upregulation of BECLIN-1 and depletion of lysosomal membrane protein 2 (LAMP2) [145]. BECLIN-1 is a core component of the lipid-kinase BECLIN-1-PI3KC3 complex that regulates autophagosome nucleation and maturation [146], while LAMP2 maintains lysosomal stability and promotes autophagic flux [147]. In such an unbalanced condition, autophagy is not only ineffective, it promotes cardiomyocyte death and increases cardiac injury [148].

Nowadays, it is clear that autophagy induced by IPC or iPostC is essential for cardioprotection [149,150]. Of note, IPC upregulates autophagy in the myocardium by enhancing the expression of the autophagosomal membrane-specific proteins LC3-II, BECLIN-1, and B-cell lymphoma-2 (BCL-2)-associated athanogene (BAG-1), a multifunctional pro-survival molecule that binds with BCL-2 and protects cells from apoptosis [151]. Huang et al. (2010) observed that IPC increases the number of autophagosomes in the risk zone of preconditioned hearts and upregulates the expression of the autophagy marker p62 in both the mCherry-LC3 transgenic mice and in the Langendorff model [152], while Velez et al. (2016) reported that IPC modulates autophagy by increasing the LC3-II/LC3-I ratio and enhancing the degradation of p62 in autolysosomes, which is related to the maintenance of the autophagic flux [29].

On the other hand, iPostC attenuates myocardial I/R injury by activating autophagy via the AMPK/endothelial nitric oxide synthase (eNOS) signaling pathway in murine models and in H9c2 cells exposed to H/R [153,154]. In particular, iPostC increases the formation of autophagic vacuoles and triggers the expression of LC3-II, BECLIN-1, LAMP2 and cathepsin D, while inhibiting the expression of p62 [153,154]. Remarkably, the study by Gua et al. (2015) revealed that iPostC regulates autophagic activity in a time-dependent manner; it is increased during the first hours and repressed from 12–24 h following iPostC in rats subjected to coronary occlusion [155]. The dual effect of iPostC could be used to propose novel therapeutic interventions that modulate autophagy as a function of post-reperfusion time.

Other pharmacological approaches have been tested to determine the cardioprotective role of autophagy. For example, the protection conferred by preconditioning with the A1 adenosine receptor agonist, 2-chloro-N(6)-cyclopentyl-adenosine (CCPA) [156], sulfaphenazole [157], is lost in the presence of the dominant-negative inhibitor of autophagy, Tat-ATG5^K130R^. In particular, sulfaphenazole preconditioning triggers autophagy through the activation of protein kinase C delta (PKCδ), and its autophagy-mediated protective effects are abolished with chelerythrine, a PKC inhibitor [157]. Additionally, simvastatin preconditioning, a lipid-lowering statin that inhibits 3-hydroxy- 3-methylglutaryl coenzyme A (HMG-CoA) reductase, induces autophagy in HL-1 cardiomyocytes and mice subjected to I/R. This treatment enhanced LC3-II and p62 expression levels and increased autophagic flux, along with the inhibition of the RAC-alpha serine/threonine-protein kinase (AKT)/mTOR signaling [158]. Simvastatin treatment is known to prevent the activation of AKT, mTOR, ULK and the ribosomal subunit protein S6. On the contrary, supplementation with mevalonate, the HMG-CoA reductase product, or the knockdown of ULK1 inhibits, statin-mediated attenuation of AKT/mTOR signaling and blocks autophagy induction [158]. On the other hand, berbamine postconditioning improves myocardial performance and cell survival through regulation of autophagy by preventing I/R-induced impairment of autophagosome processing and recovering autophagic flux in perfused rat hearts and isolated cardiomyocytes. Mechanistically, berbamine postconditioning reduced the expression of LC3-II, p62 and BECLIN-1 through activation of the phosphatidylinositol 3-kinase (PI3K)/AKT signaling pathway [150].

Sevoflurane has been widely explored as a cardioprotective molecule and recent data place it at the forefront of regulatory MQC mechanisms, particularly autophagy and mitophagy. In this respect, sevoflurane preconditioning (SPC) confers delayed cardioprotection against myocardial infarction in rats and isolated guinea pig hearts. SPC attenuates the inflammatory response and apoptosis [159,160,161] and promotes autophagosome formation, restores impaired autophagic flux and upregulates LC3-II, LAMP2 and cathepsin B (a lysosomal cysteine peptidase required for autolysosome maturation), while it downregulates p62 and BECLIN-1 in rat and guinea pig hearts. Conversely, SPostC reduces excessive autophagic activation and restores autophagic flux by downregulating PI3KC3 VPS34, reducing the interaction of VPS34 with BECLIN-1 and preventing the formation of BECLIN-1/VPS34 complex that is required for the induction of PI3KC3 activity and activation of autophagy [12,37,38,39,40]. In contrast, this anesthetic upregulates BCL-2 levels to promote the formation of the BCL-2/BECLIN-1 complex, which modulates the crosstalk between autophagy and apoptosis. In addition, SPostC decreases the number of autophagic vacuoles and promotes clearance of the autophagosome by inhibiting the I/R-induced elevated expressions of LC3-II/I, BECLIN-1, p62, ATG5 and ATG7, as well as by promoting lysosomal function through increased expression and activity of cathepsin B [12,37,38,39,40]. As the effects of SPostC on autophagy are blocked by the lysosomotropic agent chloroquine or the non-specific nitric oxide synthase (NOS) inhibitor, NG-nitro-L-arginine methyl ester (L-NAME), it follows that activation of NOS and the production of NO are indispensable in the regulation of autophagy by SPostC [37].

Moreover, a recent study demonstrated that the combination of SPostC, micro-RNA (miR)-206 (miR-206) inhibitor, AMPK activator AICAR suppresses oxidative stress, autophagy and apoptosis in rats with I/R myocardial lesions. The inhibition of miR-206 activates the AMPK pathway, modulates the expression of apoptotic proteins BCL-2 and BCL-2-associated X protein (BAX) and restrains autophagy. mir-206 together with SPostC and AICAR decrease the size of myocardial infarction and cardiac muscle fiber disorders [40]. On the other hand, SPostC prevents H/R-induced apoptosis of cardiomyocytes by inhibiting autophagy in human-induced pluripotent stem cell-derived cardiomyocytes by inactivating PI3KC3, which limits the formation of BECLIN-1/PI3KC3 complex that is essential for autophagosome formation [162], while in a simulated I/R model, SPostC protects neonatal rat cardiomyocytes by reducing apoptosis and autophagy via the PI3K/AKT signaling pathway. Finally, SPostC combined with miR-208a inhibitor or the PI3K/AKT pathway activator improves cardiomyocyte cell viability [163].

## 7. Mitophagy: The Selective Pathway to Remove Dysfunctional Mitochondria

### 7.1. Mitophagy

Mitochondrial autophagy, so-called mitophagy, is a key cellular process that selectively removes damaged mitochondria or unwanted mitochondria for subsequent lysosomal degradation, limiting ROS production and preventing the spread of damage to neighboring mitochondria and the opening of mPTP [8]. In addition, this process regulates mitochondrial homeostasis and maintains an appropriate number of healthy organelles in the mitochondrial pool [164].

Mitophagy activation depends on the processes regulated by: (1) PINK1/Parkin and (2) BCL-2/adenovirus E1B 19 kDa protein-interacting protein 3 (BNIP3)/BNIP3-like (BNIP3L, also called NIX). The first pathway involves dimerization and auto-phosphorylation of the serine/threonine kinase PINK1 on the OMM upon ΔΨm disruption [165]. Its activation promotes the recruitment and phosphorylation of the E3 ubiquitin ligase Parkin, and subsequent Parkin-mediated ubiquitination of OMM proteins, such as voltage-dependent anion channel 1 (VDAC1), to form poly-ubiquitin chains that are phosphorylated by PINK1 [166]. At the same time, Parkin recruits p62 to the poly-ubiquitinated chain to bridge both proteins with the mitochondrial ubiquitin and the LC3 on the phagophore to initiate mitophagosome formation [167]. On the other hand, loss of ΔΨm and ROS production triggers mitophagy by the BNIP3/NIX pathway [168]. In such conditions, dimerization of the mitophagy receptors BNIP3 and NIX are essential for the recruitment of autophagic machinery into the OMM [169], allowing their interaction with LC3 to engulf damaged mitochondria into mitophagosomes [170]. In addition to BNIP3 and NIX, several autophagy receptors direct programmed mitochondrial elimination in the OMM, such as FUN14 domain containing 1 (FUNDC1) [171], optineurin (OPTN), and nuclear dot protein 52 kDa (NDP52) [172], as well as prohibitin 2 (PHB2) found in the inner mitochondrial membrane, which is required for PINK1/Parkin-induced mitophagy [173,174].

### 7.2. Regulation of Mitophagy by Cardiac Conditioning

Mitophagy is activated in cardiomyocytes subjected to H/R as a cardioprotective defense, but it also constitutes a double-edged sword driving cardiomyocytes to cell death [175,176]. In fact, growing evidence establishes that mitophagy deregulation induces mitochondrial damage, resulting in mitochondrial genome collapse, electron transport chain complex inhibition, mitochondrial biogenesis arrest, cardiolipin oxidation, oxidative stress, mPTP opening, mitochondrial debris accumulation and eventually mitochondrial apoptosis [8]. In this regard, PINK1 or Parkin overexpression protects cardiomyocytes against simulated I/R injury by decreasing their susceptibility to mPTP opening [177,178], while the deficiency of PINK1 or Parkin in knockout models makes hearts more vulnerable to I/R injury, reducing survival and developing larger infarcts associated with reduced mitophagy and increment of dysfunctional mitochondria [177,178]. Conversely, BNIP3 activation enhances myocardial I/R injury, promotes mitochondrial fragmentation and mediates mitochondrial permeabilization favoring apoptosis, but at the same time, it upregulates mitophagy, promoting the conversion of LC3-I to LC3-II and the accumulation of autophagosomes containing mitochondria [179,180].

Ma et al. (2012) demonstrated that the stimulation of mitophagy through the forced expression of transcription factor EB (TFEB), a lysosomal biogenesis factor, increases lysosome numbers, restores autophagosome processing and attenuates BNIP3-induced cardiomyocyte death [181]. Additionally, the antiapoptotic dual-specificity protein phosphatase 1 (DUSP1) provides protective effects against I/R injury by preventing mitochondrial damage and cellular apoptosis via the suppression of the MFF-required mitochondrial fission and inhibiting excessive BNIP3-related mitophagy through the inactivation of the c-Jun N-terminal kinase (JNK) pathway. In contrast, DUSP1 ablation contributes to cardiac dysfunction [182]. Alternatively, overexpression of polo-like kinase 1 (PLK1) alleviates H/R-induced apoptosis and promotes the expression of mitophagy-related proteins by inducing the AMPK/FUNDC1 pathway in H9c2 cells, while the inhibition of FUNDC1 abolishes the protection [183]. On the contrary, mammalian STE20-like kinase 1 (MST1) is upregulated in response to I/R injury, repressing the MAPK/extracellular signal-regulated kinase (ERK)-cAMP-response element binding protein (CREB) pathway and consequently inhibiting cardioprotective FUNDC1-mediated mitophagy. Thus, MST1 reduces the LC3-II expression and increases the LC3-I content, as a result of faulty mitophagosome synthesis that contributes to cell death, myocardial inflammation and contractile dysfunction in mouse hearts subjected to LAD occlusion [51]. It has also been described that the MAPK/ERK-CREB pathway preferentially activates mitophagy in various disease models, such as cerebral I/R injury [184]. Likewise, MST1 repressed the expression of mitochondrial LC3-II, ATG5, BECLIN-1, VPS34 and FUNDC1 in cardiomyocytes after H/R stress. Nevertheless, the genetic ablation of MST1 in mice and cardiomyocytes sustains cardiac function, prevents mitochondrial dysfunction, ameliorates apoptosis and supports mitophagy, whereas the knockdown of FUNDC1 blocks mitophagy [51].Additionally, mitochondrial protein WD repeat-containing protein 26 (WDR26) is induced by both ischemia and hypoxia. Interestingly, WDR26 overexpression protects H9c2 cells by promoting Parkin translocation to mitochondria and facilitating hypoxia-mediated mitophagy [185].

On the other hand, mitochondrial aldehyde dehydrogenase 2 (ALDH2), a protective enzyme that metabolizes acetaldehydes, toxic aldehydes and ROS, also regulates mitophagy by suppressing the activation of the PINK1/Parkin pathway and preventing oxidative stress in H/R-treated H9c2 cells [186]. Moreover, the glycolytic enzyme glyceraldehyde-3-phosphate dehydrogenase (GAPDH) regulates the balance between cell survival by mitophagy and cell death in response to H/R. Inactive GAPDH associates with mitochondria and promotes the direct uptake of damaged mitochondria into lysosomal-like structures for elimination. GAPDH activation by PKCδ prevents its association with mitochondria, favoring the accumulation of damaged mitochondria and apoptosis. Accordingly, PKCδ inhibition or GAPDH mutation at its phosphorylation site rescues mitophagy [187].

IPC induces the selective degradation of mitochondria by mitophagy [188]. IPC promotes the translocation of Parkin to depolarized mitochondria in isolated perfused hearts and in vivo in mice. Moreover, Parkin is essential for IPC-induced p62 recruitment to mitochondria, since p62 translocation is absent in Parkin knockout mice. What is more, Parkin protein deficiency eliminates the cardioprotection by IPC, and results in increased susceptibility to myocardial infarction [178,188]. Interestingly, uncoupling protein 2 (UCP2), which is an IMM protein involved in uncoupling OXPHOS from ATP synthesis, contributes to the cardioprotective effects of IPC by enhancing I/R-activated mitophagy [189]. Furthermore, UCP2 overexpression mimicked IPC-induced protection, in association with LC3-II overexpression and downregulation of p62, TIM23 and TOM20 via the PINK1/Parkin signaling pathway. As expected, the knockdown of UCP2 nullifies the cardioprotection and mitophagy induced by IPC [189]. It is predictable that iPostC regulates mitophagy, but the mechanisms involved are unknown. Some evidence supporting this argument comes from studies where remote ischemic postconditioning (RIPostC) inhibits the overactivation of mitophagy [190,191], or those in which Hyp-PostC and RIPostC promote mitophagy through the PINK1/Parkin pathway [192], providing neuroprotective effects against ischemia and hypoxia.

Pharmacologic preconditioning with simvastatin confers cardioprotection to I/R through induction of mitophagy in HL-1 cells and mice hearts with LAD occlusion [158]. In particular, simvastatin inhibits the synthesis of ubiquinone, coenzyme Q (CoQ), which is required for transferring electrons from complex I to complex III. In consequence, it reduces ROS production, impairs OXPHOS, decreases mitochondrial membrane potential, enhances fission, and triggers the translocation of Parkin and p62 to the mitochondria. However, simvastatin cardioprotection via Parkin-mediated mitophagy is blocked by the administration of CoQ [158]. Notably, donepezil pre- and postconditioning improves autophagic and mitophagic processes by restoring LC3-II, PINK1 and Parkin expression, which results in reduced apoptosis [126]. Melatonin pretreatment induces mitophagy by upregulating LC3-II, BECLIN-1, VPS34 and TIM23, as well as promoting the interaction between LC3-II and mitochondria, as was observed by immunofluorescence [131]. Conversely, melatonin postconditioning inhibits excessive mitophagy in H9c2 cells subjected to anoxia/reoxygenation [130].

On the other hand, triiodothyronine (T_3_) postconditioning (THPostC) ameliorates the I/R-induced loss of ΔΨm and exacerbates cell death in rat hearts and cardiomyocytes by stimulating mitophagy through the PINK1/Parkin pathway. THPostC increased the expression of LC3-II, PINK1 and PARKIN and repressed the protein levels of p62, TOM20 and TIM23. That the cardioprotective effects of thyroid hormone depend on mitophagy regulation was demonstrated by inhibiting autophagy with Mdivi-1 or silencing PINK1 and Parkin [193]. No less interesting are the results obtained by enhancing the secretion of the endogenous opioid peptide enkephalin through plasmid vectors in hearts subjected to I/R injury. It was reported that overexpression of preproenkephalin reduces myocardial I/R injury in rats, diminishes mitochondrial damages, improves mitochondria ultrastructure and enhances mitophagy by upregulating PINK1, Parkin and LC3 [194].

Finally, SPostC protects against I/R injury by attenuating mitochondrial fragmentation and inhibiting the consequent mitophagy through the increment of OPA1 expression and decreasing DRP1 and Parkin levels. Interestingly, autophagosomes containing mitochondria were observed by transmission electron microscopy in the myocardial tissue of I/R rats; however, SPostC stimulated autophagic clearance by decreasing the number of autophagosomes [12]. In contrast, Yang et al. (2019) identified that SPostC promotes mitophagy through the hypoxia-inducible factor-1 (HIF-1)/BNIP3 signaling pathway to attenuate H/R injury in H9c2 cardiomyocytes, since the inhibition of HIF-1 or silencing BNIP3 increase mitochondrial damage, swelling and apoptosis, along with the accumulation of autophagosomes [195].

Conditioning strategies must maintain an adequate balance between the activation/inhibition of mitophagy to avoid eliminating healthy mitochondria and preventing energy imbalance, as well as the death of the cardiomyocyte. Therefore, it will be interesting to evaluate the progression of mitophagy over time in response to myocardial conditioning.

## 8. Mitochondria-Dependent Cell Death: A Team Work Sacrifice

Cardiomyocyte death during I/R is driven by the mitochondrial-dependent processes of necrosis, apoptosis, pyroptosis and necroptosis. The molecular entities or signaling components activated in many of these pathways are intercommunicated and although it is generally thought that membrane permeabilization characteristic of necrotic death occurs subsequently to the activation of other types of cell death, it is nowadays recognized that various cell death types occur simultaneously.

The distinctive features of necrosis, e.g., oxidative stress, Ca^2+^ overload and consequent mPTP opening contribute to apoptosis through membrane permeabilization and cytochrome C release in reperfused hearts [196]. For a long time, necrosis has been considered a passive and unregulated type of cellular death, constituting the major contributor for cardiomyocyte loss in reperfused myocardium; however, cell suicide programs, collectively defined as regulated cell death pathways, also contribute to cardiac damage and dysfunction [197]. Among them, apoptosis has been envisioned as a possible target for therapeutic manipulation, as it is a tightly regulated pathway. However, controversy on its actual participation in cardiomyocyte cell death during I/R has persisted until now [198]. A clear example of such doubt are the results obtained with the selective inhibitor of caspase-3, (S)-(+)-5-(1-(2-methoxymethyl-pyrrolidinyl)sulfonyl)isatin, which diminishes infarct size and apoptotic cell death in isolated rabbit hearts [199], while other groups described that the same compound improves cardiac function in rats independently of the regulation of apoptosis [200,201]. In between, a relatively new emergent route has been described that shares characteristics of both types of cell death called “programmed necrosis” or “necroptosis” [202] that could be as relevant as the cellular quality control processes in which organelles, proteins and lipids are recycled. Necrosis is mainly related to ischemic damage in cardiomyocytes, whereas apoptosis coexists with necrosis [203], necroptosis, and other mechanisms of cell death during reperfusion in other cardiac cells [204]. Fibroblasts, endothelial cells, and leucocytes outnumber cardiomyocytes in the heart, affecting cardiomyocyte survival and contractile performance [205]. Therefore, apoptosis and other regulated cell death pathways still can be regarded as possible targets in cardioprotection. In this sense, it has been proposed that the modification of redox-sensitive cysteine residues in members of the caspase family might be considered as a point of apoptosis regulation [206].

### 8.1. Necrosis, Apoptosis, Necroptosis and Pyroptosis Mechanisms

#### 8.1.1. Necrosis

This irreversible cell death process is accompanied by loss of cellular membrane potential, cell swelling, rupture and inflammation. Mitochondrial Ca^2+^ overload triggers mPTP opening, which causes extensive dissipation of the proton gradient across the inner membrane, inhibiting ATP synthesis and producing electron transport chain dysfunction.

mPTP, first described in the 1990s, was depicted to be composed of ANT and sometimes the mitochondrial phosphate carrier (PiC), VDAC and cyclophilin D (CypD) in the matrix [207]. However, controversy on its molecular composition persists to date. For example, even for the notion that ANT is capable of forming pores in artificial membranes [208] and that its specific ligands, bongkrekic acid and atractyloside, regulate mPTP opening [209], knockout/knockdown studies have challenged this assumption, as mitochondria from ANT1/2 double null mouse liver still had mPTP activity [210]. Other mitochondrial proteins, including some F_1_-F_0_ ATPase subunits, have been pointed out as the pore-forming core of the mPTP as determined from RNA interference-mediated depletion of the oligomycin sensitivity-conferring protein (OSCP) and isoforms of the c-subunit of the F_0_ [211]. However, ANT, PiC and CypD have not been discarded as critical inducers of Ca^2+^-induced mPTP opening.

#### 8.1.2. Apoptosis

Mitochondria-driven apoptosis or intrinsic apoptosis is related to the activation and translocation of the pore-forming proteins BCL-2 antagonist/killer (BAK) and BAX to the OMM, favoring the release of pro-apoptotic factors, e.g., cytochrome C, second mitochondria-derived activator of caspase (SMAC)/direct inhibitors of apoptosis (IAP)-binding protein with low pI (DIABLO), endonucleases, apoptosis inductor factor (AIF) and OMI/HtrA2 to the cytosol [212]. Cytochrome C is bound to the apoptotic protease activating factor-1 (Apaf-1) that binds procaspase-9 through their CARD (caspase recruitment domain) forming the apoptosome, in which the zymogen is autocleavaged to the active form caspase-9, which finally activates the executioner caspase-3. Once released from mitochondria, the proteins SMAC/DIABLO and HtrA2/Omi bind to IAPs, eliminating their suppressive effects on caspase activity and promoting apoptosis [213]. Finally, AIF is translocated to the nucleus, causing DNA fragmentation and chromatin condensation. Among the new players in the apoptotic pathway is the novel caspase recruitment domain protein 9 (CARD9), which inhibits cardiomyocyte apoptosis by interacting with Apaf-1 and blocks apoptosome formation in conditions of oxidative stress. Additionally, CARD9−/− mice have increased apoptosis after I/R [214].

#### 8.1.3. Necroptosis

Necroptosis was defined as a pathway that resembles necrosis and is an alternative to apoptosis, based on evidence that death receptor activation in the presence of caspase inhibitors conduce to cell death [215]. Necroptosis has morphological characteristics of oncosis, with intact nuclei and plasmatic membrane permeabilization. In response to ligand binding, tumor necrosis factor receptor 1 (TNRF-1) trimerizes and binds to the TNFR1-associated death domain protein (TRADD) along with other cytosolic proteins, such as the receptor interaction protein kinase-1 (RIPK1) and proteins that inhibit apoptosis, forming an assembly called complex I [216]. Complex I, without TNRF-1 but containing caspase-8 and adaptor Fas-associated death domain (FADD) protein, constitutes complex IIa, which could drive cell death to apoptosis or to necrosis if caspase-8 is inhibited [217]. Next, RIPK1 binds and activates the receptor interacting protein kinase-3 (RIPK3) comprising the necrosome, which in turn phosphorylates the mixed lineage kinase domain-like pseudokinase (MLKL), leading to downstream signaling events of programmed necrosis [218]. Phosphoglycerate mutase 5 (PGAM5) is a serine/threonine-protein phosphatase located in the OMM, which has been thought to be a necroptotic regulatory factor that favors the assembly of RIPK3-MLKL [219]. The idea that necroptosis is a mechanism related to ROS production [220] and mitochondrial transition pore activation [221,222] has been maintained since this term was first used. A discussion of early studies that sustain this hypothesis can be found in other reviews [223].

More recent evidence has been obtained from mouse microvascular endothelial cells in which necroptosis linked to RIPK1/RIPK3 activation was partially inhibited by cyclosporin A (CsA), a well-known inhibitor of mPTP opening [222]. Additionally, in a cardiomyocyte model that mimics animal reperfusion, the contribution of the mitochondrial mPTP opening rate as an upstream trigger of cellular necroptosis was evaluated, and it was demonstrated that RIPK3 produces ER stress, Ca^2+^ overload, xanthine oxidase activation, ROS production and consequently mPTP opening [224]. The regulatory action of the protein sarcoendoplasmic reticulum calcium transport ATPase (SERCA) and the mitochondrial Ca^2+^ homeostasis on necroptosis has also been evaluated using spermine, a mitochondrial Ca^2+^ uniporter agonist in cardiac microvascular endothelial cells subjected to H/R. SERCA overexpression prevented both RIPK3 and mitochondrial phosphatase PGAM5 upregulation, whereas spermine suppresses this effect, promoting mitochondrial Ca^2+^ overload and opening of the mPTP [225]. However, other authors have shown that necroptosis is not a mitochondrial-dependent pathway and that ROS-dependent opening of the mPTP is not directly related to cell death [224]. A recent report shows that cardiomyocyte-restricted ablation of Cops8, an essential subunit of the ubiquitination regulator of necroptosis, causes increased RIPK1-RIPK3 interaction along with oxidative stress. Nevertheless, ablation of CypD (the mPTP inhibitory regulator) did not decrease, but rather augmented necrosis and premature death in Cops8^CKO^ mice, indicating that necroptosis was independent of mPTP opening [226]. It remains to be answered whether mitochondrial components or ROS derivatives are executors of the necroptosis program, and to discard whether it is a problem of a particular cell type or experimental conditions, and such information should be obtained from more complex systems.

#### 8.1.4. Pyroptosis

This is a pro-inflammatory cell death program activated after the interaction of proteins with damage-associated molecular patterns (DAMPs) that lead to the formation of the intracellular NOD-, LRR- and pyrin domain-containing protein 3 (NLRP3) inflammasome complex, which promotes autoproteolytic cleavage and consequent activation of caspase-1 [227]. This protease activates gasdermin D (GSDMD) that forms plasmatic membrane pores, cell lysis and the so-called pyroptotic death [228,229]. Following inflammasome activation, NLRP3 is recruited to mitochondria by its interaction with an OMM adaptor named mitochondrial anti-viral signaling protein (MAVS) and with cardiolipin [230]. Thereby, although pyroptosis ultimately causes cell death from creating pores in the plasmatic membrane, dysfunctional mitochondria could contribute to the assembly and activation of the NLRP3 inflammasome through ROS-mediated processes, involving the binding of oxidized mtDNA to NLRP3 and subsequent activation of caspase-1. Such findings have been reported mainly in immune cells, whereas in cardiac cells and particularly in reperfusion damage, this issue remains unclear and requires further experimental support. In this sense, a recent report showed that myocardial infarct size, cardiac function, and mitochondrial morphology were significantly improved in a rat model of I/R after hydrogen inhalation, along with diminution of 8-hydroxydeoxyguanosine (8-OHdG), ROS and NLRP3 [231].

### 8.2. Regulation of Mitochondrial-Dependent Cell Death by Cardiac Conditioning

#### 8.2.1. The Main Target of Reperfusion Damage: The mPTP

The clear relationship between necrotic death in reperfusion damage and the opening of the mPTP has been envisioned for a long time as a main target in cardioprotection. To date, due to the recently reported changes in the molecular identity of the pore and the relatively new discovery of the Ca^2+^ uniporter structure, which together with ROS are the main regulators of the opening of this pore, novel inhibitors are currently being tested.

One of the most studied targets in the mPTP is the prolyl isomerase CypD, the mPTP regulator located in the matrix. CsA is considered a bona fide CypD ligand [232]; in vitro it inhibits mitochondrial swelling and increases mitochondrial Ca^2+^ retention capacity, in vivo it reduces infarct size in preclinical studies, but its translation into clinical trials has failed [233]. However, this has not been the end of the story, as the post-translational regulation of mPTP by phosphorylation and S-acylation of CsA is receiving special attention. Preventing CypD phosphorylation at S191 by inhibiting the glycogen synthase kinase-3β (GSK3β) reduces the translocation and binding of CypD to OSCP, avoiding increased ROS production, mPTP opening and subsequent cell death at reperfusion [234]. Moreover, it has been demonstrated that C202 in CypD is S-acylated and that ischemia induces deacylation and Ca^2+^ overload. Additionally, this cysteine is S-nitrosylated in IPC [235]; therefore, when C202 was mutated into C202S, which cannot undergo post-translational modifications, mice subjected to I/R showed less injury and resistance to mPTP opening and diminished binding to the ATP synthase, suggesting that a free cysteine is needed to target CypD to the mPTP [236].

HK2 is also a well-known regulator of mPTP opening. Accordingly, the cell-permeable cAMP analog 8-Br has recently been shown to protect the heart against regional I/R injury at the onset of reperfusion by promoting the binding of HK2 to mitochondria and inhibiting mPTP, as measured by the reduction in Ca^2+^-induced mitochondria swelling [237]. This study contrasts with the one reported by Pereira et al. (2020), in which in vitro dissociation of mt-HK2 from mitochondria has no effect on mPTP opening in postconditioned hearts [120]. This group concluded that mt-HK2 inhibition of the pore is not directly associated with HK2 binding and that it could rather be related to the binding of KH-II to the mitochondrial fission proteins, DRP1 and DRP2. Therefore, the observed changes in cristae structure and mitochondrial morphology might destabilize contact points in mitochondria promoting cytochrome C release and sensitizing mPTP to Ca^2+^ overload. Once the identity of the mPTP can be fully established, there is no doubt that a huge step would be made to prevent necrotic death; meanwhile, the search for new CypD inhibitors continues.

#### 8.2.2. Inhibiting the Mitochondrial Apoptotic Program

A hallmark of the mitochondrial apoptotic pathway is cytochrome C release, which may occur in combination with necrosis due to mitochondrial swelling or after the formation of BAX/BAK pores. Both pharmacological strategies [238] and mechanical maneuvers [239] have shown to diminish apoptotic cell death in correlation with increased BCL-2/BAX ratios and diminished extramitochondrial cytochrome C levels. The upstream regulation of this pathway involves dephosphorylation of VDAC1 through the PI3K-AKT-GSK3β pathway. Antioxidants [240], α2-adrenergic receptor agonists [241] and volatile anesthetics have strong cardioprotective effects, which are related to PI3K-AKT signaling [242]. Phospho-AKT inactivates GSK3β, reducing VDAC1 phosphorylation, favoring BAX dissociation from mitochondrial membranes, and inducing VDAC1/HK2 binding, resulting in reduced ROS generation, inhibition of cytochrome C release and reduced apoptosis. For example, resveratrol preconditioning activates and promotes the translocation of GSK-3β from the cytosol to mitochondria, where it interacts with CypD and prevents mPTP opening [243]. Additionally, long-term nutritional preconditioning with resveratrol inhibited VDAC1 expression induced by I/R and attenuated mitochondria-mediated apoptosis [244].

Crosstalk between apoptotic and mitophagy regulatory elements has been described. The cellular location where such interactions occur determines whether the fate of the cells will progress to one or the other process. BNIP3, FUNDC1 and NIX, are expressed in the OMM, while PARKIN translocates to mitochondria in response to low membrane potential [245]. Additionally, the mitochondrial anti-apoptotic protein BCL-2 regulates the autophagic process by interacting with BECLIN-1 at the OMM. Protection against I/R damage by the phenolic compound Paenol is related not only carried out with its antioxidant and anti-inflammatory properties, but with increased expression of BCL-2 and downregulation of BECLIN-1, BAX, LC3, and p62, sustaining the hypothesis of crosstalk between apoptosis and autophagy [246]. Additionally, it has been shown that vitamin D, MitoTEMPO or Mdivi-1 treatments reduced COX4 colocalization with LC3 (autophagosomes) in cardiomyocytes subjected to H/R [179].

The role of mitophagy in reperfusion injury is paradoxical, as the opposite to apoptosis is considered cardioprotective in I/R injury; however, excessive mitochondrial clearance might also reduce healthy mitochondrial number, unbalancing cellular energy production and promoting ROS production and cardiac damage.

#### 8.2.3. Controlling Necroptosis and Pyroptosis

In 2005, Degterev et al. described a potent and selective necroptosis inhibitor named necrostatin-1 (Nec-1) that targets RIPK1 and stabilizes its inactive conformation [215]; very soon after, this and other groups confirmed the cardioprotective effect of the tryptophan-based small molecule in several models of myocardial I/R damage [247]. Nec-1 also inhibits MLKL recruitment into the necroptosome, reduces RIPK3 and PGAM5, decreases mitochondrial fission and maintains ΔΨm [248]. The excitement around Nec-1 has been hampered by reports showing that intracoronary injection of the compound produced a positive inotropic effect, but did not reduced infarct size [249] and also that at certain doses it produces cardiotoxicity [250].

The RIPK3–MLKL axis is fundamental for the execution of necroptosis in the plasma membrane and, consequently, is a plausible target in cardioprotection. However, its role in mitochondria is not that clear and has become a controversial issue. It has been reported that neither the canonical RIPK3–MLKL pathway nor the proposed non-canonical molecular axis, PGAM5–DRP1 and JNK–BNIP3, are activated in an ex vivo model of reperfusion damage. Even then, RIPK3 inhibition with GSK’872 or HS-1371 prevented plasma membrane rupture and delayed mPTP opening; the association found with this effect was an increase in manganese superoxide dismutase (MnSOD) expression, suggesting that RIPK3 modulates oxidative stress [251]. On the other hand, SPostC has been shown to reduce RIPK1/RIPK3/MLKL-mediated necroptosis, increase RIPK3 O-GlcNAcylation and diminish necroptosis both in an in vivo model and in isolated hearts subjected to I/R [252].

There are scarce data on cardioprotective strategies directed to regulate pyroptosis during reperfusion damage. FK866, a nicotinamide phosphoribosyltransferase inhibitor, which binds to Toll-like receptor 4 (TLR4) and activates the inflammasome [253], was evaluated to determine its efficacy against brain reperfusion damage in rats subjected to cardiac arrest/cardiopulmonary resuscitation. FK866 inhibited the activation of NLRP3, downregulated the expression of NLRP3, interleukin-1 beta (IL-1β), GSDMD and p-DRP1, improving mitochondrial morphology [254]. More recently, it has been reported that hydrogen gas inhalation after 24 h after reperfusion in an in vivo rat model inhibits oxidative stress and decreases NLRP3 expression [231]. There are no experimental reports that attempt to inhibit the association of oxidized mtDNA with the inflammasome in order to demonstrate mitochondrial participation in the pyroptotic pathway; however, indirect evidence exists, as such interactions activate downstream caspase-1, placing it at a critical point of regulation. A recent example is the utilization of the compound VX-765 that reduced infarct size, lactate dehydrogenase release and preserved mitochondrial complex I activity [254], a response mainly driven by fibroblasts that express the inflammasome-caspase-1 axis [255].

To date, the extent to which these pathways actually contribute to myocardial death during reperfusion damage has not been fully determined. What is clear is that necroptosis and apoptosis, activated by different stimuli, are cell death pathways specifically dependent on mitochondria.

## 9. The Role of EVs in Cardiac Conditioning and MQC Mechanisms

### 9.1. EVs: Microvesicles, Exosomes and Apoptotic Bodies

EVs, including microvesicles, exosomes and apoptotic bodies, are lipid membrane-enclosed vesicular structures secreted by cells into the extracellular space under cellular activation or stress, which are present in biological fluids, such as blood, urine and saliva [256]. Microvesicles are ∼100–1000 nm in diameter and are formed directly from the outward budding of the plasma membrane [257]; exosomes are formed by the endosomal route and are the smallest of the vesicles, measuring ∼30–150 nm [258], whereas apoptotic bodies range from 100–5000 nm and are produced from cells undergoing apoptosis [259]. EVs act as biological carriers and mediators of intercellular communications by delivering their content and surface proteins to different types of cells [260]. In this manner, uptake of EVs can modulate the function and phenotype of target cells [261]. These vesicles carry a variety of cargo, including RNAs, genomic DNA, proteins, free fatty acids and lipids [262]. Interestingly, exosomes, regardless of their small size, can incorporate intact mitochondria [263] and deliver cardiolipin, mtDNA and some mitochondrial proteins [264].

Circulating EVs have been related to cardiovascular diseases as well as with cardiac protection [265,266], constituting potential diagnostic biomarkers. Therefore, EVs have drawn attention in recent years to establish novel strategies to prevent heart damage after myocardial infarction.

### 9.2. Exosomes-Delivery to Protect Heart against Reperfusion Injury

Plasma exosomes isolated from the blood of rats and humans promote cardioprotection in different experimental models of I/R injury [267]. For example, HL-1 cells pretreated with rat exosomes and exposed to H/R showed resistance to mitochondrial depolarization and cell death. Similar results were obtained in ex vivo and in vivo models, in which the infusion of plasma exosomes decreased infarct size following I/R. It has been demonstrated that heat shock protein 70 (HSP70) located on the exosome surface mediates cardioprotection by activating pro-survival signaling pathways, including TLR4, ERK1/2 and p38 MAPK, which phosphorylate HSP27. They confirmed that neutralization of HSP70 or TLR4 and kinases inhibition avoids cardiac protection [267]. Remarkably, HSP70 is a key component of mitochondrial import, translocation and protein folding [268], which are essential functions for mitochondrial biogenesis.

Mesenchymal stem cells (MSCs) are multipotent stromal cells that can differentiate into a variety of cell types, improving ischemic heart function due to their potent regenerative effects [269]. MSCs can differentiate into cardiomyocytes, endothelial cells, pericytes, and vascular smooth muscle cells, restoring the damaged myocardium [270]. Specifically, MSCs-derived exosomes have been suggested to act as cell-free vectors that show low immunogenicity, high biocompatibility and are capable of conferring cardioprotection and promoting revascularization and regeneration in target cardiac cells, contributing to survival and a reduction in myocardial fibrosis [271,272]. Bone marrow-MSCs-derived exosomes overexpressing the proinflammatory cytokine macrophage migration inhibitory factor (MIF) restored cardiac function, and reduced heart remodeling, mitochondrial fragmentation, ROS generation and apoptosis in a rat model of myocardial injury [273]. Experimental studies of myocardial infarction have described that after IPC application, MSCs can excrete miR-22-enriched exosomes, which are transferred to cardiomyocytes, reducing myocardial apoptosis [274], and additionally, such particles prevent mitochondrial fission through AMPK signaling [273]. Another interesting piece of information found in the scientific literature revealed that MSCs-derived EVs protect kidneys from I/R injury by restoring mitochondrial dynamic balance, inhibiting fission and reducing apoptosis [275].

A recent study in vitro determined that miR-210 loaded on endothelial progenitor cells (EPCs)-derived exosomes protects endothelial cells from H/R damage [276]. In particular, miR-210 alleviates oxidative stress-associated cardiomyocyte apoptosis by regulating BNIP3 [277]; therefore, EPCs exosomes overexpressing miR-210 attenuate angiogenic dysfunction, decrease apoptosis and mitochondrial fragmentation, increase ΔΨm and ATP levels, as well as regulate mitochondrial dynamics [276]. Additionally, exosomes from adipose-derived stromal cells (ADSCs) enriched with miR-93-5p attenuate inflammation, preventing cardiac injury caused by myocardial infarction [278]. miRNAs contained in ADSCs-derived exosomes promote angiogenesis and decrease apoptosis, inflammation, and fibrosis after an ischemic insult [279], while miR-146a-containing exosomes alleviated fibrosis, inflammation, and apoptosis after myocardial ischemia by downregulating the transcription factor early growth response factor 1 (EGR1) [280]. At this point, it is worth mentioning that a single injection of small EVs of energetically stressed adipocytes limits cardiac I/R injury in mice, introducing oxidatively damaged mitochondria, which enter circulation and are taken up by cardiomyocytes, inducing a burst of ROS in cardiac tissue. This pro-oxidant signal resembles IPC conditions that promote protection against oxidative stress [281].

On the other hand, cardiosphere-derived cells (CDCs) possess cardiac regenerative properties and improve function in the infarcted rat heart [282]. Remarkably, CDCs-secreted exosomes delivered to infarcted pig hearts reduce scarring, attenuate adverse remodeling, and improve function after myocardial infarction [283]. Furthermore, CDCs-derived exosomes increase cardiomyocyte survival by preventing hypoxia-induced apoptosis [284]. These reports highlight the relevance of exosomes as potential cardioprotective agents. However, the clinical application of stem cell-derived exosomes must overcome technical challenges, such as the mode of delivery and a detailed content characterization [265]. In addition, the fact that EVs diminish atherosclerosis [285] and consequently lower the probability of myocardial infarction constitutes an exciting therapeutic possibility. Additionally, EVs *per se* can induce protective responses as their loaded cargo could induce survival signals and prevent mitochondrial dysfunction, triggering a conditioning-like response.

### 9.3. Pre- and Postconditioning and EVs in Cardiac Protection

IPC and iPostC strategies regulate miRs related to cardiac conduction. A reduction in miR-1, miR-208, and miR-328 levels in iPostC animals has been reported, explaining at least in part the antiarrhythmic effect of this maneuver [286]. On the other hand, human umbilical vein endothelial cells (HUVEC) subjected to IPC release exosomes to protect cardiomyocytes against H/R via the ERK1/2 signaling pathway, thus preventing cell death [11,287]. Of note, ERK1/2 activation is associated with the inhibition of mPTP opening and maintenance of mitochondrial function during reperfusion [288]. Furthermore, intramyocardial injection immediately after coronary occlusion of serum exosomes from rats that underwent cardiac IPC into infarcted naïve rat hearts induced cardiac protection and limited the infarct size by decreasing inflammation and apoptosis via the activation of the BCL-2 and PI3K/AKT pathway, along with downregulation of caspase-3 and BAX [289]. Similarly, IPC microvesicles were collected and applied via the femoral vein to naïve rats subjected to coronary occlusion. IPC microvesicles decreased infarct size, improved cardiac function, reduced apoptosis and inhibited ER stress. Mechanistically, IPC microvesicles upregulate BCL-2, inhibit cleavage of caspase-3, as well as downregulate BAX and the ER stress sensors, glucose regulatory protein 78 (GRP78), C/EBP homologous protein (CHOP) and caspase-12 [290,291]. In this regard, it is well recognized the role of BCL-2/BAX ratio and caspase-3 in the intrinsic apoptosis pathway, whereas BCL-2 is also involved in mitochondrial dynamics [292].

Long non-coding RNA (lncRNA) contained in exosomes also participates in cardioprotection. For example, the urothelial carcinoma-associated 1 (lncRNA-UCA1) was detected in exosomes obtained after applying HypC in human MSCs (HypC-MSCs). Such exosomes were delivered into infarcted rat hearts, conferring cardioprotection and predictably, lncRNA-UCA1 has been pointed out as a key protective mediator and biomarker in heart diseases [293]. In another study, exosomes enriched with miR-125b also obtained from HypC-MSCs triggered heart protection in a mouse model of myocardial infarction [294], whereas exosomes/microvesicles from cardiac fibroblasts exerted protection in cardiomyocytes subjected to H/R. Hyp-PostC enhanced protection via miR-423-3p upregulation in the vesicles [295].

Mitochondrial dysfunction is tightly associated with myocardial reperfusion injury. Up to now, we have found data that EVs are implicated indirectly in the maintenance of mitochondrial function and MQC mechanisms in hearts following myocardial infarction. Intercellular communication provided by EVs could allow proper signaling to prevent mitochondrial dysfunction for cardiac healing after myocardial infarction. However, there is still a need to study the role of mitochondria in the delivery of cardiac EVs in conditioning strategies. In this regard, a recent study showed that intramyocardial injection of mitochondria-enriched EVs after myocardial infarction was able to improve heart function in mice, suggesting that mitochondrial transfer or bioenergetic cargo can contribute to maintain the endogenous mitochondrial network and contribute to alleviate heart damage in a conditioning-like effect [263].

### 9.4. Remote Conditioning and EVs in Cardiac Protection

RIC-derived EVs are also a promising therapeutic strategy to improve cardiac cell remodeling and function after myocardial infarction. In this respect, Jeanneteau et al. (2012) suggested that “circulating microparticles” link RIC and cardioprotection [296]. Previously, Giricz et al. (2014) suggested that EVs released from the heart after IPC were necessary to transfer RIPC cardioprotective signaling [297]. Later, exosomes isolated from RIC rats were shown to promote cardiac cell remodeling and angiogenesis following artery occlusion by targeting HSP70. This finding supports the results obtained by RIPC and RIPostC on cardiac function after myocardial infarction and confirms the importance of vesicular transfer mechanisms in remote cardioprotection [298]. In addition, RIC-derived EVs from human plasma transferred cardioprotection and reduced infarct size when EVs were perfused in rat hearts subjected to I/R injury ex vivo. These benefits were associated with the upregulation of miRNAs that target the mTOR pathway, including miR-16-5p, miR-144-3p and miR-451a [299]. Additionally, RIC-derived EVs from serum of patients under isoflurane anesthesia protected H9c2 cardiomyoblasts against hypoxia-evoked apoptosis [300].

Likewise, RIC application in a rat model inhibits cardiac dysfunction, oxidative stress, and maladaptive remodeling through exosome-transmitting signals that included the antifibrotic factor miR-29, contributing to heart failure prevention [301]. Conversely, RIPC upregulates miR-144 in the mouse myocardium and increases miR-144 levels in plasma exosomes of mice and humans, protecting the heart and augmenting cardiomyocyte survival in response to I/R injury by activating AKT, GSK3β, and ERK1/2 signaling, as well as decreasing mTOR level and autophagy [302]. Although these data do not have direct effects on mitochondria, they certainly have indirect implications. For instance, miR-144 overexpression stimulated mitochondrial biogenesis via AMPK activation and deacetylation of PGC-1α to finally attenuate apoptosis, protecting the heart from hyperglycemia-induced injury [303]. In addition, the elevated expression of the anti-apoptotic miR-24 was identified in plasma exosomes from rats subjected to RIPC. More important, RIPC exosomes transport miR-24 to protect naïve rat hearts and H9c2 cardiomyocytes from myocardial I/R injury, decreasing myocardial apoptosis by downregulating the expression of pro-apoptotic protein BIM [304]. On the other hand, transfusion of platelet-derived microvesicles from rats that underwent RIPC to open chest-operated rats undergoing I/R, attenuates heart infarction and improves cardiac function [305].

## 10. Conclusions and Future Directions

**Figure 3:**
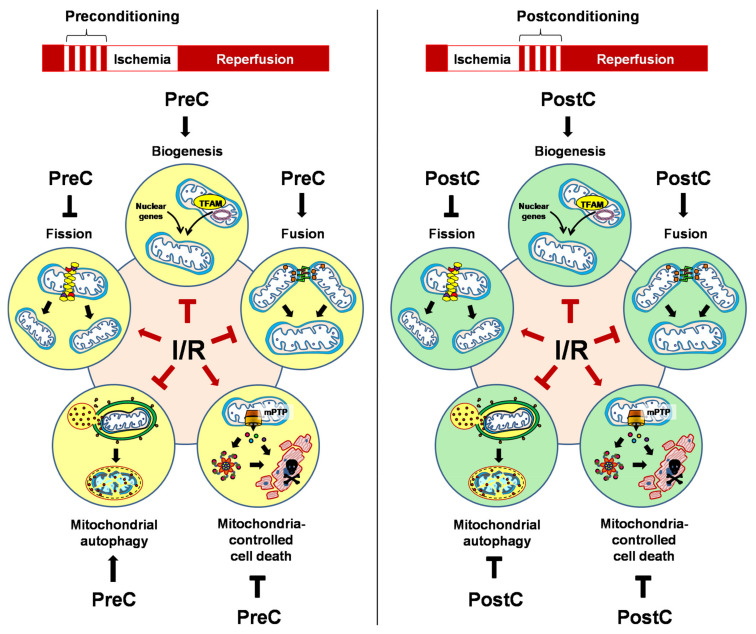
Myocardial conditioning regulates mitochondrial quality control (MQC) processes disturbed by ischemia/reperfusion (I/R) injury. In general, myocardial preconditioning (PreC) strategies promote mitochondrial biogenesis, fusion and mitophagy, while inhibiting fission and mitochondria-controlled cell death. On the other hand, myocardial postconditioning (PostC) strategies induce mitochondrial biogenesis and fusion; while attenuate fission, mitophagy and mitochondria-controlled cell death. TFAM, mitochondrial transcription factor A; mPTP, mitochondrial permeability transition pore.

Cardioprotective strategies effectively modulate MQC mechanisms and counteract alterations caused by I/R injury. Pre- and postconditioning strategies positively regulate mitochondrial biogenesis by activating AMPK and inducing PGC-1α upregulation, leading to the replacement of damaged mitochondria. Moreover, both strategies reestablish the balance in mitochondrial dynamics by positively regulating fusion through the activation of OPA1 and MFN2, whereas they negatively regulate fission by inhibiting DRP1, preventing mitochondrial fragmentation. Autophagy and mitophagy are triggered by pre- and postconditioning strategies. In particular, mitophagy is activated via the PINK1/Parkin pathway to eliminate depolarized mitochondria. Nonetheless, postconditioning also inhibits excessive autophagy and mitophagy in order to prevent cell death. Consequently, both strategies negatively regulate mitochondria-dependent cell death (Figure 3). Despite the growing interest in MQC mechanisms, the role of IPC and IPostC in the regulation of mitochondrial biogenesis, dynamics, mitophagy, necroptosis and pyroptosis remains poorly understood and requires more exploration. Finally, the modulation of MQC mechanisms and the recognition of potential mitochondrial targets that are susceptible to being regulated by conditioning strategies could provide potential and selective therapeutic approaches for I/R-induced mitochondrial dysfunction.

On the other hand, it is increasingly evident that EVs play a key role in cardiac conditioning. EVs protect the heart against I/R injury by transferring cardioprotective molecules to cardiomyocytes. However, few data correlate EV delivery with cardiac MQC mechanisms and mitochondrial protection; therefore, it will be relevant to delve into this topic. In association, mitochondria-derived vesicles (MDVs) have emerged as another means of interorganellar communication and their relationship to cardiac MQC processes has only recently been revealed [306]. Although limited, the evidence indicates that MDVs could constitute a defense mechanism against cardiac damage, making it mandatory to propose new research which recognizes their relevance for cardioprotection during I/R injury and their potential modulation by conditioning strategies.

Additionally, a quite new mechanism of intermitochondrial communication has been described, which involves the formation of specialized double-membrane protrusions, termed mitochondrial nanotunnels; moreover, it was suggested that such structures provide a means of matrix content exchange and functional complementation similar to that achieved by mitochondrial fusion [307]. However, the role of mitochondrial nanotunnels in MQC has not been explored in depth, much less its involvement in cardioprotection; therefore, it will be crucial to analyze these mechanisms in future studies.

On the other hand, mitochondrial transfer from cell to cell through EVs, Cx43 gap junctions or tunneling nanotubes can provide a mitochondrial source for replenishing dysfunctional mitochondria [308], and it should be included as a prospective MQC mechanism. Further studies are required to identify whether cardioprotective strategies can trigger the transfer of mitochondrial content between cells of cardiac tissue in response to myocardial infarction.

## Data Availability

Not applicable.
